# Supplementary material for: A broad-spectrum gas sensor based on correlated two-dimensional electron gas
Source: Nat Commun. 2023 Dec 21;14:8496. doi: 10.1038/s41467-023-44331-7 (PMC10739975; doi:10.1038/s41467-023-44331-7)
Supplement: Supplementary file 1 — SUPPLEMENTARY INFO [file 41467_2023_44331_MOESM1_ESM.pdf]

Supplementary Materials for

A Broad-spectrum Gas Sensor based on Correlated Two-Dimensional Electron Gas

Yuhao Hong[1], Long Wei[1], Qinghua Zhang[2], Zhixiong Deng[1], Xiaxia Liao[3], Yangbo Zhou[3], Lei Wang[1], Tongrui Li[1], Junhua Liu[1], Wen Xiao[1], Shilin Hu[1], Lingfei Wang[4], Lin Li[1], Mark Huijben[5], Kai Chen[1], Gertjan Koster[5], Guus Rijnders[5*], Zhaoliang Liao[1,5*]

[1]*National Synchrotron Radiation Laboratory, University of Science and Technology of China; Hefei, 230029, China.*

[2]*Beijing National Laboratory for Condensed Matter Physics, Institute of Physics, Chinese Academy of Sciences; Beijing, 100190, China.*

[3]*School of Physics and Materials Science, Nanchang University; Nanchang, 330031, China.*

[4]*National Research Center for Physical Sciences at Microscale, University of Science and Technology of China; Hefei, 230026, China.*

[5]*MESA+ Institute for Nanotechnology, University of Twente; Enschede, 7500 AE, the Netherlands.*

*To whom correspondence should be addressed: a.j.h.m.rijnders@utwente.nl, zliao@ustc.edu.cn

1. Growth of $LaFeO_3$ and $LaAlO_3$ films

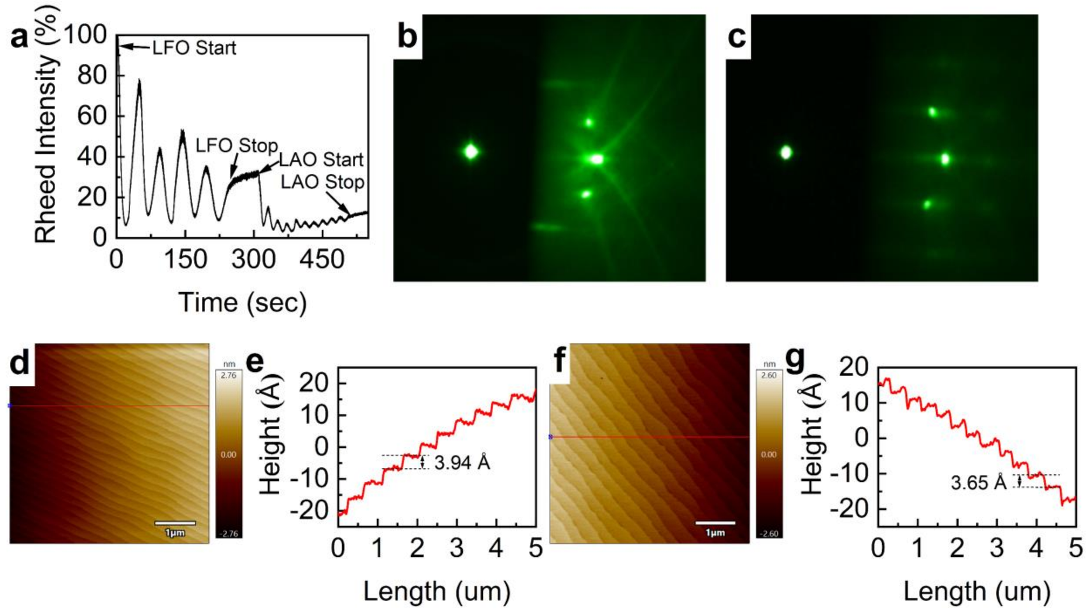

Fig. S1 | Growth and surface characterization. a, RHEED intensity oscillations during the growth of LFO (5 u.c.) and LAO (10 u.c.). The growth started at t = 0 and stopped at the time indicated by arrows. RHEED patterns before **(b)** and post **(c)** growth. AFM surface topography before **(d)** and post **(f)** growth. Measured step heights before **(e)** and post **(g)** growth.

The $LaFeO_3$ (LFO) and $LaAlO_3$ (LAO) films were grown by pulsed laser deposition (PLD) in a layer by layer fashion as shown in Fig. S1a. The thickness of the LFO and LAO was controlled by counting the RHEED oscillations, enabling a precise unit cell (u.c.) control of the growth. RHEED patterns (See Fig. S1, b-c) indicate a 2D smooth surface. The AFM surface topography (Fig. S1d) and measured step heights (Fig. S1e) before growth indicate that a high-quality single TiO_2-terminated $SrTiO_3$ (001) substrates were obtained. Highly smooth film AFM surface image (Fig. S1f) shows that the grown film still has a two-dimensional structure, and measured step heights (Fig. S1g) of thin films shows that the LAO is subjected to tensile strain.

2. X-ray diffraction and reflectivity of heterojunction film

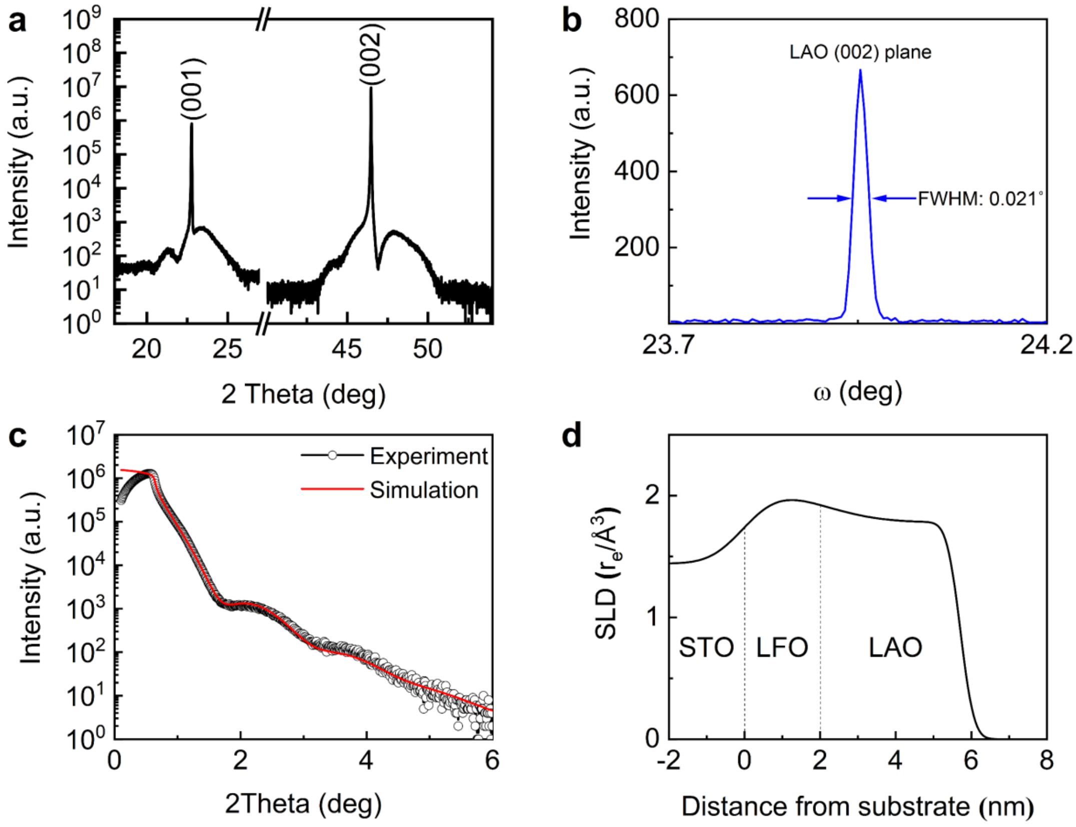

Fig. S2 | Structure characterizations. a, X-ray diffraction of (001) and (002) peaks for the heterojunction film. **b**, X-ray diffraction ω-rocking curve of the as-grown heterojunction film (002) plane. Experimental (black dots) and model (red line) X-ray reflectivity curves **(c)** of the heterojunction film. **d**, X-ray scattering length density (SLD) profiles obtained from the X-ray reflectivity fits.

X-ray diffraction (Fig. S2a) shows that the heterojunction film has a good epitaxial relationship with the STO substrate. The full-width at half-maximum (FWHM, Fig. S2b) was measured to be 0.021°, indicating the good crystallinity of the heterojunction film. The simulation of XRR (Fig. S2c, red line) well confirm the layer by layer fashion monitored by RHEED. The simulation parameters are shown in Table S1.

3. X-ray absorption

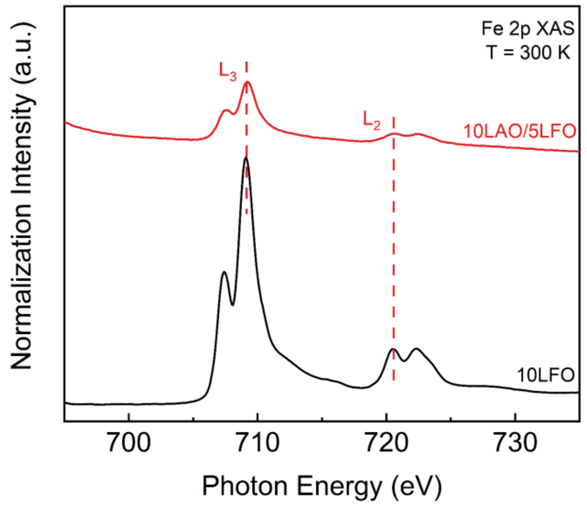

Fig. S3 | X-Ray absorption spectroscopy (XAS) of Fe L-edge (Fe 2p). The XAS of the Fe L-edge of the heterojunction (red line, $10LaAlO_3/5LaFeO_3/SrTiO_3$) and the single $LaFeO_3$ film (black line, $10LaFeO_3/SrTiO_3$).

Since the capped LAO layer blocks the release of excited electrons, the electron yield decreases, leading to a reduction of total electron yield signal and thus intensity in the underneath Fe XAS. However, the XAS results do not show an evident change of valence state of Fe.

4. Detections of low boiling point and inert substances

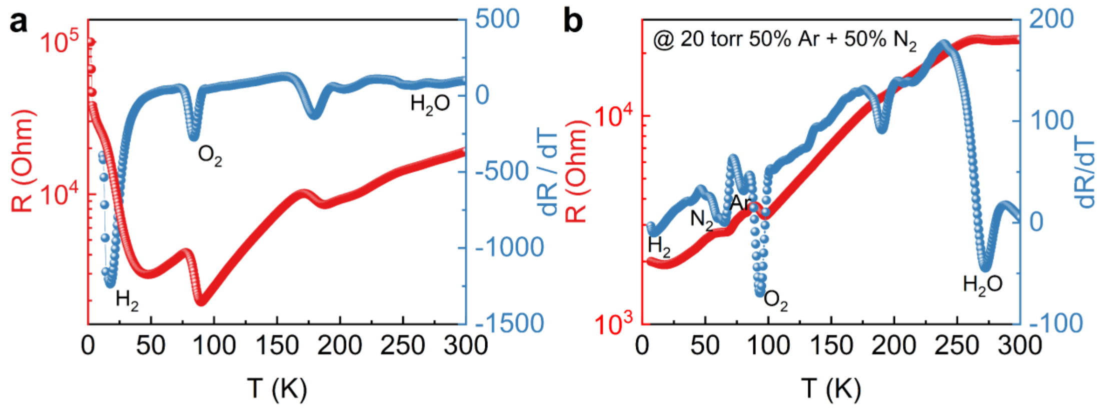

Fig. S4 | Optimized activation resistance for broader gas detection. Measurements of H_2 **(a)** and Ar **(b)**.

By adjusting the carrier concentration at the interface of C-2DEG gas sensor, the resistance after activation does not exceed the measurement limit of the source meter, so as to realize the detection of those substances that sublime or boil at low temperature, such as H_2 (Fig. S3a) and Ar (Fig. S3b).

5. Evidence for Charged Gas Ice layer

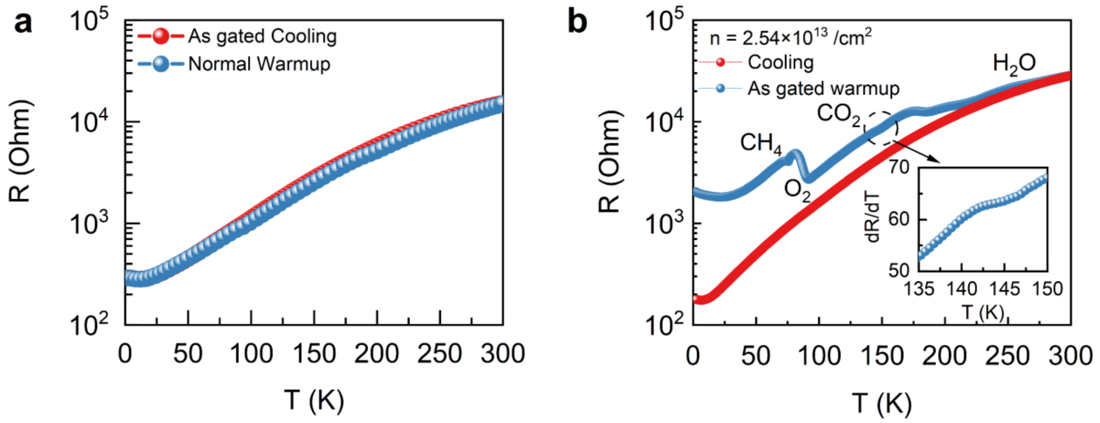

Fig. S5 | Evidences for Charged gas ice layer. A, R-T curves of cooling (red line) and warmup (blue line) with gate applied at 300K. **b,** Response of sample with higher carrier concentration (2.54×10^{13} /cm^2) in mixed gas environment. The inset is the first order differential of resistance around the CO_2 sublimation temperature (135 K - 150 K).

Two smooth curves (Fig. S4a) show that applying back-gating at high temperature (300K) does not activate the device, but only the gas ice layer can be charged. The sudden change of the slope of the first differential (Fig. S4b) indicates the sublimation of CO_2. Based on the parallel plate series model ($1/C=1/C_1+1/C_2$), the total capacitance is mainly contributed by the substrate since the film thickness is much thinner than the substrate. Under the temperature of 2 K and the electric field of 4200 V/cm, the STO (001) substrate has a relative permittivity of ~10^4.[1] Therefore, after applying a back-gate voltage of 210 V, the gas charge concentration is ~2.32×10^{13}/cm^2, which is consistent with the depleted interface carrier concentration calculated in the main text.

6. Characterization of carrier mobility before and after activation

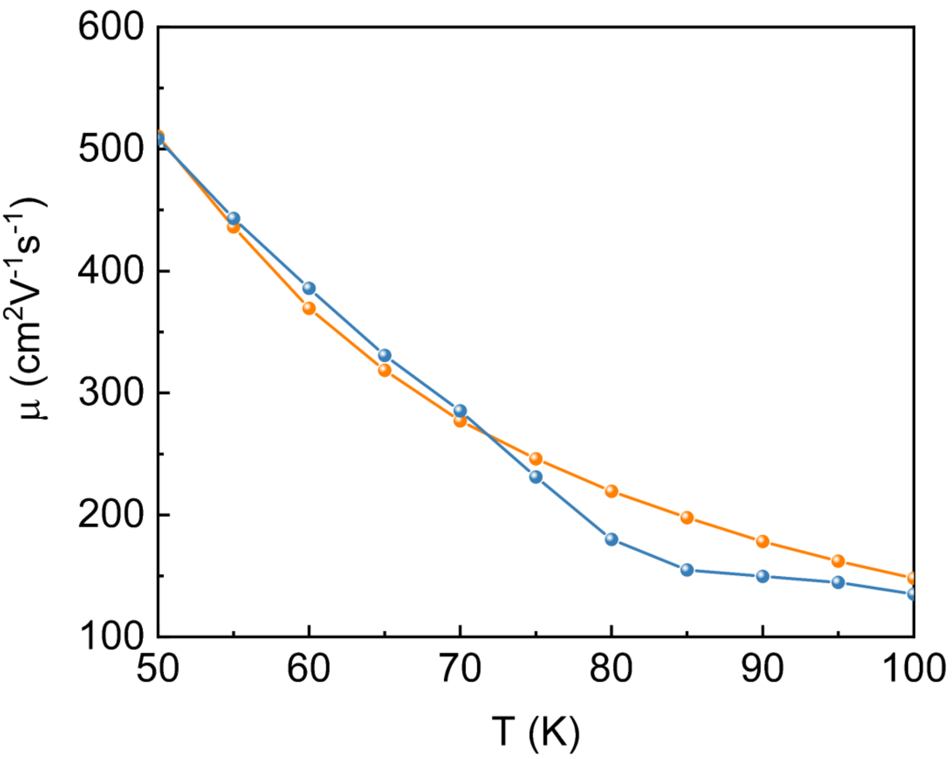

Fig. S6 | Carrier mobility before (orange line) and after (blue line) activation as a function of temperature.

The carrier mobility after activation is almost the same as that of as grown, except that there is a maximum 20% change in the SOB temperature range, indicating that the activation does not change the carrier mobility, but only reduces the carrier concentration.

7. Characterization of devices with different carrier

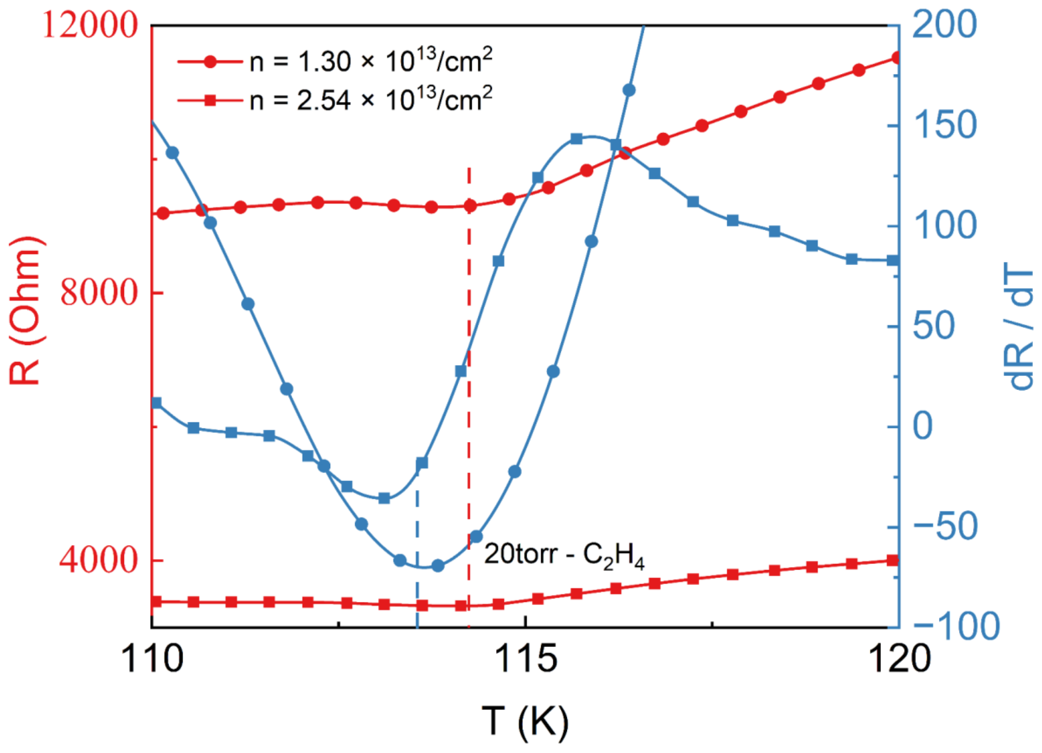

Fig. S6 | Effect of devices with different carrier concentrations on temperature.

The circular markers correspond to the sensor with a carrier concentration of 1.30 × $10^{13}/cm^2$, while the square markers correspond to the sensor with a carrier concentration of 2.54 × 10^{13} $/cm^2$. The temperature difference associated with both the inflection points and the minimum value of the first-order differential of the two sensor resistances is less than 0.5 K within the error bars.

Layer	*Chem. Formula*	*Density (g/cm^3)*	*Thickness (Å)*	*Roughness (Å)*
Layer 2	$LaAlO_3$	6.822347	37.22338	2.835766
Layer 1	$LaFeO_3$	7.998116	19.9194	12.57356
Substrate	$SrTiO_3$	5.236075		7.181943

Table S1. The simulation parameters obtained from the X-ray reflectivity fits by GenX 3.6.16. Thickness fitting results of LFO and LAO are consistent with STEM images (Fig. 1c).

Name	*CO_2 measure limit*	*Compatibility*	*Working temperature*	*Response time*	*Author*
C-2DEG Gas Senor	0.025 torr (33 ppm at 1.0 atm)	Broad-Spectrum	-273 °C – Room temperature	30 - 600 s (Depends on heating rate)	Present work 2023
Calcium doped ZnO	50 ppm	H_2 & CO	350 °C	111 s	Ghosh et al. 2019[2]
p-Si/MoO_3	100 ppm	Unknown	250 °C	8 s	T. Thomas et al. 2021[3]
$Li_4Ti_5O_{12}$	100 ppm	Unknown	500 °C	5 s	S. Joshi et al. 2016[4]
$CoAl_2O_4$	100 ppm	CO	450 °C	50 s	C. Michel et al. 2010[5]
ZnO	400 ppm	H_2 & CO	350 °C	75s	Y. Hunge et al. 2018[6]
CeO_2	800 ppm	H_2 & CO	250 °C	Unknown	A. Aboud et al. 2017[7]
SnO_2	2000 ppm	Unknown	240 °C	350 s	D. Wang et al. 2016[8]
CN_x/p-Si	3 torr	Unknown	Room Temperature	260 s	N. Zouadi et al. 2015[9]

Table S2. A literature review on the performance parameters and conditions of CO_2 gas sensor.

According to the parallel plate capacitor model, the device exhibits an instantaneous response when a substance undergoes sublimation or boiling. which is manifested as a change in the slope of the C-2DEG resistance at the interface. To precisely characterize gas partial pressure, data should be acquired within a range of ±10 K around the sublimation or boiling point. Consequently, the response time for measuring partial pressure depends on the heating rate (2 - 40 K/min). As a result, the response time can range anywhere from 30 to 600 s.

Compounds	*Electron Affinity (eV)*
H_2 (g)	0.75[10]
N_2 (g)	0.07[11]
CH_4 (g)	-1.63[12]
O_2 (g)	1.46[13]
C_2H_4 (g)	-0.38[12]
CO_2 (g)	0.6±0.2[14]
ice	0.8±0.1[15]
Ar (g)	-1

Table S3. A literature review on the electron affinity of all gases appearing in the article. The higher the electron affinity, the stronger the ability to bind electrons, the more sensitive our gas sensors are to this gas and thus have a higher measurement limit.

Reference

1 Neville, R. C., Hoeneisen, B. & Mead, C. A. Permittivity of Strontium Titanate. *Journal of Applied Physics* **43**, 2124-2131, doi:10.1063/1.1661463 (1972).

2 Ghosh, A., Zhang, C., Shi, S. & Zhang, H. High temperature CO_2 sensing and its cross-sensitivity towards H_2 and CO gas using calcium doped ZnO thin film coated langasite SAW sensor. *Sensors and Actuators B: Chemical* **301**, doi:10.1016/j.snb.2019.126958 (2019).

3 Thomas, T. *et al.* Porous silicon/α-MoO_3 nanohybrid based fast and highly sensitive CO_2 gas sensors. *Vacuum* **184**, doi:10.1016/j.vacuum.2020.109983 (2021).

4 Joshi, S., Lanka, S., Ippolito, S. J., Bhargava, S. K. & Sunkara, M. V. {111} faceted $Li_4Ti_5O_{12}$ octahedra as the reference electrode material in a nanostructured potentiometric CO_2 sensor. *Journal of Materials Chemistry A* **4**, 16418-16431 (2016).

5 Michel, C. R. CO and CO_2 gas sensing properties of mesoporous $CoAl_2O_4$. *Sensors and Actuators B: Chemical* **147**, 635-641, doi:10.1016/j.snb.2010.04.013 (2010).

6 Hunge, Y. M., Yadav, A. A., Kulkarni, S. B. & Mathe, V. L. A multifunctional ZnO thin film based devices for photoelectrocatalytic degradation of terephthalic acid and CO_2 gas sensing applications. *Sensors and Actuators B: Chemical* **274**, 1-9, doi:10.1016/j.snb.2018.07.117 (2018).

7 Aboud, A. A. *et al.* CO_2 responses based on pure and doped CeO2 nano-pellets. *Journal of Materials Research and Technology* **7**, 14-20, doi:https://doi.org/10.1016/j.jmrt.2017.03.003 (2018).

8 Wang, D. *et al.* CO_2-sensing properties and mechanism of nano-SnO_2 thick-film sensor. *Sensors and Actuators B: Chemical* **227**, 73-84, doi:https://doi.org/10.1016/j.snb.2015.12.025 (2016).

9 Zouadi, N., Messaci, S., Sam, S., Bradai, D. & Gabouze, N. CO_2 detection with CNx thin films deposited on porous silicon. *Materials Science in Semiconductor Processing* **29**, 367-371, doi:10.1016/j.mssp.2014.07.023 (2015).

10 McWeeny, R. The electron affinity of H_2: a valence bond study. *Journal of Molecular Structure: THEOCHEM* **261**, 403-413 (1992).

11 Haynes, W. M. *CRC handbook of chemistry and physics*. (CRC press, 2016).

12 Hedges, R. M. & Matsen, F. A. Antisymmetrized Hückel Orbital Calculations of Ionization Potentials and Electron Affinities of Some Aromatic Hydrocarbons. *The Journal of Chemical Physics* **28**, 950-953, doi:10.1063/1.1744302 (1958).

13 Kristiansson, M. K. *et al.* High-precision electron affinity of oxygen. *Nat Commun* **13**, 5906, doi:10.1038/s41467-022-33438-y (2022).

14 Yin, W. J. *et al.* The Effect of Excess Electron and hole on CO_2 Adsorption and Activation on Rutile (110) surface. *Sci Rep* **6**, 23298, doi:10.1038/srep23298 (2016).

15 Gaiduk, A. P., Pham, T. A., Govoni, M., Paesani, F. & Galli, G. Electron affinity of liquid water. *Nat Commun* **9**, 247, doi:10.1038/s41467-017-02673-z (2018).
